# Meckelin guides orientation of basal bodies along the striated rootlet

**DOI:** 10.1186/2046-2530-4-S1-P67

**Published:** 2015-07-13

**Authors:** T Picariello, M Valentine, A Nabi, J Yano, J Van Houten

**Affiliations:** 1 Dept. of Biology, University of Vermont, Burlington, VT, United States

## Objective

Meckelin (MKS3) functions in ciliogenesis and ciliary gating. MKS3 appears to have similar functions in *Paramecium tetraurelia*, i.e. FLAG-MKS3 is found associated slightly above each basal body and RNAi for *MKS3 *leads to loss of cilia. RNAi for *MKS3 *also leads to the disorganization of rows of basal bodies that run from anterior to posterior. In areas of misalignments, basal bodies with their post ciliary and transverse rootlets are found out of their expected rows. However, the rootlets are attached to the basal bodies at the expected angles relative to each other.

We propose that MKS3 guides new basal bodies as they move toward the anterior of the cell along the striated rootlet (SR) of the parent basal body. Basal bodies without MKS3 lose their interactions with the parent's SR. Without this guide to maintain orientation, new basal bodies migrate off the expected line and, when they form their SRs, these too cannot project toward the anterior as expected.

## Method

We tagged 13 potential SR components and examined their location. Nine were associated with the SR, often with non-uniform distributions.

## Results

Those sequences with SF assemblin domains (similar to those in the *Chlamydomonas *rootlet proteins) coded for proteins that we found in the *Paramecium *SRs; those without this domain were not in the rootlets.

MKS3 interacted sufficiently with the epitope tagged striated rootlet proteins to be pulled down with a GST-fusion of the 252 C-terminal residues of the *Paramecium *MKS3.

## Conclusion

MKS3 interacts with a subset of SR proteins.

